# Patient Derived Models to Study Head and Neck Cancer Radiation Response

**DOI:** 10.3390/cancers12020419

**Published:** 2020-02-12

**Authors:** Pippa F. Cosper, Lindsey Abel, Yong-Syu Lee, Cristina Paz, Saakshi Kaushik, Kwangok P. Nickel, Roxana Alexandridis, Jacob G. Scott, Justine Y. Bruce, Randall J. Kimple

**Affiliations:** 1 Department of Human Oncology, University of Wisconsin School of Medicine and Public Health, Madison, WI 53705, USA; cosper@wisc.edu (P.F.C.); lindsey.abel@wisc.edu (L.A.); ylee596@wisc.edu (Y.-S.L.); cpaz@wisc.edu (C.P.); kaushik4@wisc.edu (S.K.); kpnickel@humonc.wisc.edu (K.P.N.); 2 Department of Biostatistics and Medical Informatics, UW Carbone Cancer Center, University of Wisconsin School of Medicine and Public Health, Madison, WI 53705, USA; alexandridis@biostat.wisc.edu; 3 Departments of Translational Hematology and Oncology Research and Radiation Oncology, Cleveland Clinic, Cleveland, OH 44195, USA; scottj10@ccf.org; 4 Department of Medicine, Division of Hematology and Oncology, UW Carbone Cancer Center, University of Wisconsin School of Medicine and Public Health, Madison, WI 53705, USA; jybruce@medicine.wisc.edu; 5 Department of Human Oncology, UW Carbone Cancer Center, University of Wisconsin School of Medicine and Public Health, Madison, WI 53705, USA

**Keywords:** head and neck cancer, radiation therapy, radiation, patient-derived models, cancer

## Abstract

Patient-derived model systems are important tools for studying novel anti-cancer therapies. Patient-derived xenografts (PDXs) have gained favor over the last 10 years as newer mouse strains have improved the success rate of establishing PDXs from patient biopsies. PDXs can be engrafted from head and neck cancer (HNC) samples across a wide range of cancer stages, retain the genetic features of their human source, and can be treated with both chemotherapy and radiation, allowing for clinically relevant studies. Not only do PDXs allow for the study of patient tissues in an in vivo model, they can also provide a renewable source of cancer cells for organoid cultures. Herein, we review the uses of HNC patient-derived models for radiation research, including approaches to establishing both orthotopic and heterotopic PDXs, approaches and potential pitfalls to delivering chemotherapy and radiation to these animal models, biological advantages and limitations, and alternatives to animal studies that still use patient-derived tissues.

## 1. Introduction

Patient-derived xenografts (PDXs) have become an important model system for studying novel anti-cancer therapies. First described over 70 years ago [1,2,3], PDXs have gained favor over the last 10 years as newer mouse strains, such as the NOD-*scid* IL2Rgamma^null^ (NSG) and NOD.Cg-Prkdc^scid^ Il2rg^tm1Sug^/JicTac (NOG) strains, have significantly improved the success rate of establishing PDXs from patient biopsies [4,5]. PDXs have been developed from nearly all types of human tumors and numerous groups have reported their use for testing novel therapeutics, identifying patient cohorts for precision medicine approaches, identifying biomarkers of therapeutic response, and confirming cellular mechanisms identified in vitro [6]. In our experience, PDXs can be engrafted from patient head and neck cancer (HNC) samples across a wide range of cancer stages [7,8,9,10]. Other groups using large banks of PDXs have shown that the rate of engraftment can be prognostic of poor outcomes, suggesting that PDXs may be able to provide important information about the patients most in need of novel therapies [6,11].

There are clear advantages of PDXs over other in vivo model systems: PDXs remain the only in vivo model of actual patient tumors and, at low passages, retain the genetic features of their human source; there is no sophisticated technology required; and they can provide a renewable source of cancer cells for organoid cultures. Disadvantages of this mouse model include the use of immunocompromised animals, thus eliminating potential immune-mediated anti-cancer effects, possible genetic and biological changes with increased passages, dependence on the ability of a given tumor to grow in mice to initiate studies, and the time and investment required for each additional passage. Herein, we review the uses of HNC PDXs for radiation research, including approaches to establishing PDXs, approaches and potential pitfalls to delivering radiation and chemotherapy to these animal models, and alternatives to animal studies that still use patient-derived tissues.

## 2. Patient-Derived Models of Head and Neck Cancer

### 2.1. Xenograft Models

**Figure 1:**
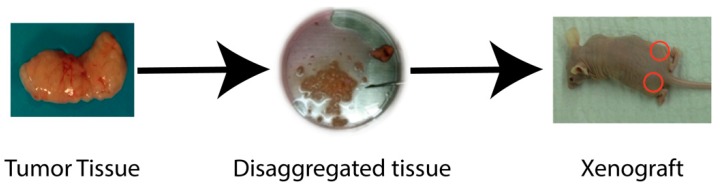
Establishment of xenografts.Tumor tissue obtained from a patient or from an animal model can be used to establish xenografts. Tissue is disaggregated under sterile conditions and implanted into the desired location of recipient mice.

**Figure 2:**
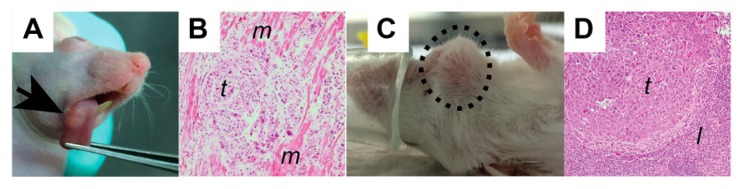
Orthotopic head and neck cancer models. (**A**) Orthotopic growth of a PDX within the tongue (arrow) can be seen with careful inspection. (**B**) On histologic evaluation the tumor (t) can be seen infiltrating into tongue muscle (m). (**C**) Lymph node metastases (dashed circle) can be seen following orthotopic tumor injection, but are less commonly seen with flank models. (**D**) Histologic evaluation demonstrates the tumor (t) within a lymph node (l).

Xenografts are a widely used cancer research model system that involves growing tumor tissue in a different species from the donor species. Xenograft models can be used to study tumor biology, investigate anti-cancer therapeutics, or for the development of predictive biomarkers. The source of cancer cells can be an established cancer cell line or dissociated patient-derived tumor tissue (Figure 1). Tumors established from syngeneic animals or cell lines (i.e., murine cancer cell line injected into a murine host), although not truly a xenograft, are also typically referred to as xenografts. Syngeneic tumors can be implanted into immunocompetent animals, while implanting tumors from one species into a second species requires the use of immunocompromised animals. When studying HNC, xenografts are most commonly injected subcutaneously into the flank of the animal (i.e., heterotopic implantation, Figure 1, right panel). However, in the past several years, some groups have taken to utilizing orthotopic xenografts in which the tumors are established in the tissue of origin (e.g., head and neck squamous carcinoma cells injected into the buccal cavity or cheek of the mouse, Figure 2) [12].

Patient-derived xenografts provide advantages over either cell-line xenografts or classical tissue culture work and can be used to predict patient response to targeted drugs [8,13,14,15,16,17]. PDXs can be established from patients with both human papilloma virus (HPV)-positive and HPV-negative head and neck squamous cell carcinomas [8,9], and from cancers with rare head and neck histologies, including adenoid cystic carcinoma and midline NUT carcinoma [7]. Importantly, individual PDXs maintain the genetic and phenotypic characteristics of the original patient tumors [6,8] including expression of viral oncogenes in HPV-positive tumors [18]. However, studies in breast cancer and acute lymphoblastic leukemia have demonstrated extensive clonal dynamics throughout serial PDX passaging [19,20]. The generalizability of this finding and its impact on therapeutic response is yet to be confirmed. Overall engraftment rates range from 50%–75% depending, at least in part, on the quantity of tumor engrafted. It has been shown in several cancers, including HNC, that PDX engraftment and growth itself is an indicator of poor prognosis, and some have suggested that PDX formation itself can be used as a risk stratification biomarker [21,22]. Other factors affecting the engraftment rate include HPV status: we have found that HPV-negative tumors seem to engraft slightly better than HPV-positive tumors, which limits our ability to study virally induced cancers in such a system. Other studies have also shown this, as well as finding that tumors with perineural invasion had a higher engraftment rate [22]. Despite this difference in the ability to establish PDXs, it appears that this does not significantly impact the biology of the resulting tumors: HPV+ PDXs are more sensitive to radiation than HPV− PDXs, consistent with what is seen clinically [8].

There are biological differences between murine and human tissues, particularly with regard to radiation response, making PDXs an attractive model. Studies performed over the years have shown that the activities and/or levels of critical molecules mediating the DNA damage response after irradiation differ between species [23]. Rodent cells are generally more susceptible to radiation-induced oncogenic transformation, less efficient at checkpoint activation, and more sensitive to oxidative stress when compared to human cells [24]. Given the differences between rodent and human cells regarding aspects of the fundamental mechanisms mediating cellular response, and that a goal of experimental radiation oncology is to develop modifiers relevant to treatment, most preclinical radiation studies focus on human tumor xenografts.

PDXs are a valuable resource for evaluating therapeutic response and for confirming putative mechanisms identified in other model systems [7,8,25,26,27,28]. Most PDXs established by our lab, and others, utilize remnant tissue provided by pathology after surgical excision of the cancer (discussed more below). This leads directly to a major limitation of using PDXs for cancer therapeutic development: we do not know how the patient would have responded to the drug, radiation, or combination therapy under investigation. Other potentially significant limitations include the presence of mouse rather than human stroma, which has implications for the tumor microenvironment.

Perhaps the most important limitation is that PDX engraftment must be performed into an immunocompromised host, precluding study of the immune response. It is now clear that the host anti-tumoral immune response is intricately involved in the overall treatment response and it has been shown that a functional immune system is required to cure HPV-positive HNC with chemoradiation (CRT) in vivo [29]. There is ample evidence that radiation therapy ignites such an immune response by increasing immunogenic antigen presentation and by releasing cytosolic DNA, which activates the cGAS/STING pathway, resulting in a type I interferon response [30]. These, and other mechanisms of radiation-induced immune activation are now burgeoning fields of research [31,32]. Thus, the lack of a functional immune system in PDX mouse models may not completely reflect how the patient’s tumor would respond to CRT. However, the development of humanized mice (see below) offers the potential to include immune studies in the context of PDXs. Though the lack of an immune system represents an obvious and clinically relevant caveat, the PDX model remains useful for studies focused on tumor-intrinsic effects, for dissecting molecular mechanisms dictating tumor cell death, and identifying resistance-promoting adaptations. Clearly, depending on the molecular target being addressed and its mechanism of action, these characteristics need to be taken into account when attempting to translate results to a clinical scenario.

### 2.2. Organoid Models

Patient-derived organoids (PDO) from different cancer types have been successfully established in recent years. In this method, isolated cells from the patient are grown as organoids in a 3D matrix, rather than in more traditional 2D culture systems, which better represent in vivo tumor architecture and cell–cell interactions. It has been demonstrated that PDOs from HNC can recapitulate the morphologic and molecular characteristics of the original tumor [33,34]. In addition, PDOs can be readily used to test sensitivity to multiple drugs and radiation due to their relatively rapid 3D culture (i.e., days), compared to the lengthy process of establishing PDXs (i.e., weeks to months) [35]. We have recently demonstrated an approach to testing radiation sensitizers using PDOs derived from a panel of colon cancer patients [36] and have active projects utilizing this approach in HNC. A major limitation of PDOs is that replication of experiments typically requires a renewable source of tissue. To address this limitation, we establish PDOs from PDX tissue, thus avoiding the need for repeated patient biopsies. To date, there is little reported about the rate at which organoids can be successfully established from PDXs. In our experience, not all tumors that grow as PDXs are able to form useful organoid cultures. Although PDOs show promise as an approach to rapidly screen patient tumors for drug response, additional validation is clearly required.

### 2.3. Zebrafish Models

Zebrafish (*Danio rerio*) have been used as model organisms in scientific research for over 50 years. These fish grow quickly, are small and inexpensive to maintain, and are easily amenable to genetic manipulation. Several groups have begun using zebrafish embryos as a host for the implantation of patient-derived tumor tissue in order to study drug sensitivity [37,38]. Drugs, including chemotherapy, can be added to the water, where they are absorbed and metabolized by the fish. If a drug is effective in eradicating the implanted tumor cells, the embryos survive, and if the drug is ineffective, the fish embryos die due to growth of the tumor cells. Major advantages of this system include the ability to screen a relatively large number of drugs in a short time period, although expertise with zebrafish is clearly necessary. One can also use zebrafish to study radiation response. Most commonly, wild-type or genetically modified embryos are irradiated and assessed for morphologic abnormalities and survival, but one can also screen radiation sensitizers using this model system [39].

### 2.4. Establishing Patient-Derived Models

Our approach to establishing PDXs was previously described in detail [8,9,10,40] and will be briefly outlined below. We obtain fresh tumor tissue from patients who consent to provide de-identified excess tissue under an Institutional Review Board (IRB)-approved protocol via our institution’s Translational Sciences Biobank (University of Wisconsin Health Sciences IRB#2016-0934, approved 10 October 2016, expiration 11 August 2020). Tissue is taken from the patient by a surgeon and placed directly into saline (not formalin!). Samples are processed by the pathology department and a portion not required for diagnostic purposes is reserved for research and provided to the research team. We transfer the tumor from saline into ice-cold Dulbecco’s Modified Eagle Medium (DMEM) and store for less than 48 h prior to use. Tissue is transferred to a sterile tissue culture dish, rinsed with sterile phosphate-buffered saline (PBS) and cleared of blood and necrotic tissue. The cancer tissue is then suspended in prepared PDX media and minced with sharp, sterile scissors to obtain a slurry. We use this slurry for implantations into animals, the establishment of organoid cultures, and/or for cryopreservation. The slurry is mixed in a 1:1 ratio with matrigel and implanted into 4–6-week-old male or female NOD-SCID gamma (NSG, NOD.Cg-*Prkdc^scid^ Il2rg^tm1Wjl^*/SzJ) mice.

### 2.5. Flank Models

Heterotopic, subcutaneous implants into the flanks of mice are advantageous because they provide easy access to the tumor site for injection, growth monitoring, and treatment. This approach is commonly used for PDX establishment due to the ease of monitoring this location and the relatively minor impact upon animal health. The tumor slurry can be slightly viscous as an 18-gauge needle can be used to implant tumor cells. Alternatively, a small incision can be made in the flank to implant small pieces of tumor, which has the advantage of initially preserving normal tumor architecture. Relatively large volumes (100–400 μL) can be implanted using a flank model which can shorten the time of tumor development compared to the orthotopic model described below. This approach also permits the growth of tumors of sufficient size to enable passage into subsequent generations, provides tissue that can be cryopreserved for later use, and/or can be used to establish PDOs. This model can also be used to monitor changes in the transplanted tumor over time by measuring tumor dimensions. One significant limitation of heterotopic HNC PDXs is that they rarely give rise to metastasis.

Following tumor cell injection, mice can be monitored weekly until tumors reach a size of at least 200 mm^3^. In our experience, this requires a minimum of 1 week and can take several months. We monitor animal weight weekly but rarely see significant changes in mouse weight due to tumor growth on the flank. To harvest tissue for analysis, mice are euthanized using Institutional Animal Care and Use Committee (IACUC)-approved protocols (University of Wisconsin IACUC protocol #M005974, approved 31 January 2018, expires 30 January 2021) and/or undergo survival surgery to remove the growing mass.

### 2.6. Orthotopic Models

Establishing orthotopic head and neck cancer PDXs requires the disaggregation of the tumor into a finely chopped suspension which is filtered using a sterile mesh in order to allow cells to be injected using a 27-gauge needle. Oweida and colleagues have demonstrated this nicely in a video method [12] that we have adopted for use with our PDXs. In our experience, any PDX that grows in the flank will grow orthotopically, although the failed implant rate (percentage of implanted sites that fail to develop tumors) is somewhat higher. A much smaller volume of cells can be injected into HNC orthotopic sites when compared to heterotopic sites (≤50 μL vs. 200–500 μL)—either the tongue, the floor of the mouth, or buccal mucosa. It is thought that this approach provides a more “natural” tumor microenvironment for tumor growth, as evidenced by the appearance of both lymph node and lung metastasis (Figure 2C). Despite the injection of smaller volumes of tissue, we have not identified systematic differences in the architecture of the resulting tumors. Given the rapidly rising incidence of HPV-associated oropharyngeal carcinomas, it would be ideal to study such orthotopic models. However, mice and other rodents lack tonsils. Their functionally equivalent tissue is nasal-associated lymphoid tissue, making this an impractical site for PDX engraftment [41].

Tumor measurements can be done using microcalipers, ultrasound, or using fluorescent or bioluminescent imaging if cells are transfected with appropriate molecular tags. Orthotopic head and neck tumors can significantly affect the animals’ ability to eat and drink, thus close monitoring of animal health and weight is mandatory. Due to the effects of the growing tumors on the animals’ oral intake, a cutoff of 20%–25% weight loss from the baseline is used as a censoring endpoint (see analysis below). This cutoff may need to be discussed with local animal care and use committees and can vary from institution to institution. The impact of orthotopic tumors on animal health has important implications for the lengths of experiments. In particular, it may be necessary to stop the less efficacious treatment arms early (e.g., control, drug alone). For this reason, in addition to monitoring tumor size, we often use survival as an endpoint with orthotopic HNC models. Though orthotopic models may more accurately recapitulate the original patient tumor, it is currently not known whether this method significantly differs from heterotopic tumor models in terms of growth rate and treatment response. This is an active area of investigation by multiple groups.

### 2.7. Humanized Models

Nearly all studies with PDXs are currently performed in immunocompromised mice. In fact, many groups, including ours, use the NSG model (NOD.Cg-*Prkdc^scid^ Il2rg^tm1Wjl^/*SzJ), which lacks B, T, and NK cells and is even more immunosuppressed than nude mice. This model system makes it impossible to study the anti-tumoral immune response or effectiveness of immunotherapies. There are genetically engineered and carcinogen-induced mouse models of head and neck cancer that are available for study in immunocompetent hosts, but they may not recapitulate the heterogeneity present in human cancers. Thus each model has advantages, but neither allows for the study of patient tumor tissue in an immunocompetent host. Humanized mouse models offer a potential solution to this problem, as they are immunocompromised to allow for PDX growth, but are then engrafted with a partially functional “human” immune system, allowing for the study of the immune response [42]. Humanized animal models offer the opportunity for investigators to study immunomodulatory agents in human cancers [43,44,45,46,47], work that has taken on greater importance since the 2019 approval of pembrolizumab in the first-line setting for metastatic HNC patients.

Humanized mice can be developed by engrafting human peripheral blood mononuclear cells (PBMCs), hematopoietic stem cells (HSC), or fetal thymus or liver tissues. Engraftment of PBMCs allows for the expansion of human T cells, however graft-versus-host disease quickly ensues, leading to the relatively short survival of the animal, which is prohibitive for long-term studies. HSC engraftment often requires pre-conditioning with irradiation or cytotoxic drugs, but newer mouse models have been developed that do not require this, and some even support the enhanced engraftment of human immune cells (i.e., MISTRG mouse) [43]. Humanized CD34+ mice are a recent advance and are created by injecting human CD34+ HSCs into NSG-recipient mice. Advantages of this model include multilineage hematopoiesis with circulating CD4+ and CD8+ T-cells with functional T-cell receptor diversity, and the ability to perform longer term studies (>12 months) due to the lack of graft-versus-host disease [48]. Despite these advances, these humanized mouse models continue to be constrained by differences in major histocompatibility complex (MHC) antigens and cytokine expression, among others, between mouse and human tissues.

We, and others, have begun to utilize humanized mouse models to study immunotherapy with PDXs [44,46,47]. At our institution, the Brown and Burlingham groups have developed a NeoThy humanized mouse model using non-fetal human tissue (i.e., cryopreserved neonatal thymus and umbilical cord blood HSC) that harbors functional human hematopoietic cells [49]. We have used NeoThy mice to successfully engraft multiple human PDXs and have demonstrated that they harbor functional immune cells (currently unpublished data).

While there are multiple approaches to engrafting human hematopoietic stem cells in mice, we believe the NeoThy approach offers significant advantages over several of the others. Notably, the NeoThy mouse is established without the use of fetal tissue. The advantage of neonatal thymus is that the lymphocytes develop a tolerance to murine tissues, which delays the onset of a graft-vs-host disease that limits the longevity of other humanized models [50,51]. This provides the ability to perform anti-cancer studies investigating long-term control of tumor growth or with tumors that grow slowly. This approach also permits the generation of a cohort of “identical” animals, each of which receives a thymic fragment and hematopoietic cells from the same source. A significant drawback to this approach is the time required for the development of a significant human immune cell population (8–12 weeks) and the fact that two temporally distinct engraftments are unlikely to result in identical animals. Interestingly, our experience, and that of other groups, is that a tumor and a donor do not need to be human leukocyte antigen (HLA)-matched to successfully engraft tumors in humanized mice. How this impacts treatment response, and whether MHC matching improves the generalizability of data from humanized models, remains unknown.

## 3. Radiation Delivery

Radiation therapy (RT) is a critical component of the treatment plan for many HNC patients. Radiation can be delivered curatively or palliatively; for the definitive treatment of cancer or in the post-operative setting; and alone or in combination with chemotherapy. Animal models are used by many groups to study potential radiosensitizers, drugs that when combined with radiation, serve to improve the therapeutic ratio and augment the effects of radiation.

The approaches and techniques described here are applicable to any animal model: PDX, cell line xenograft, syngeneic, or even in vitro organoid models. Each approach to delivering radiation has clear benefits and limitations that we will discuss. Most importantly, it is critical to confirm the delivered dose in whatever model system is used.

**Figure 3:**
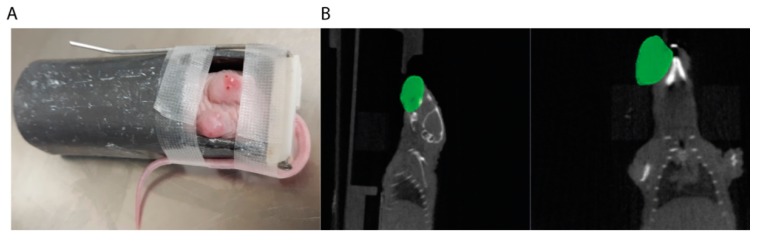
Radiation delivery to head and neck cancer (HNC) xenograft models. (**A**) Flank models, can be easily irradiated using cabinet irradiators and lead jigs. (**B**) Using an image-guided small animal irradiator, the tumor (contoured in green) can be targeted with multiple radiation beams to limit dose to other normal structures.

In recent years, we and many other groups have transitioned to using small animal irradiators capable of delivering highly conformal radiation therapy that mimics radiation delivery in the clinic. These systems (e.g., Xstrahl’s small animal radiation research platform (SARRP) or Precision X-ray’s small animal radiation therapy platform (SmART)) utilize image guidance and treatment planning software to allow users to develop individualized radiation treatment plans for each subject [52]. These systems allow the delivery of radiation with fewer side effects and toxicities of therapy. Despite these advantages, the use of these systems can have significant drawbacks as well: radiation delivery can take 5–10 min per animal and many centers require the use of specialized staff to ensure proper use of the equipment, thus increasing the costs of treatment. For these reasons and others, many groups still utilize standard cabinet or cesium irradiators with a fixed source to irradiate animal models. These systems are often capable of treating several mice at once and require almost no treatment planning time as they rely on standardized protocols, but have a limited ability to conform the radiation dose to the target. We currently tailor the radiation delivery apparatus to the model system. When performing radiation on flank xenografts, we use the cabinet irradiator with custom-built lead jigs (Figure 3A) to shield the majority of the animal from the radiation dose. This permits rapid and reproducible delivery of radiation for the majority of studies investigating radiation sensitizers that we perform in PDX models. The SARRP/SmART system is used to irradiate orthotopic models given the more complex nature of these anatomic sites (Figure 3B). While the treatment-planning software provides dose estimates, it is important to confirm dose delivery as these machines can become slightly misaligned, resulting in significant deviations in radiation dose delivery to the target.

The transition from cesium irradiators, with their easily calculated dose rate, has highlighted the importance of dosimetric validation for pre-clinical irradiation. There is currently not a standard protocol for performing calibration of irradiators used in research [53]. If they perform calibration, most facilities use a variation on the approach described in TG-61, a document describing methods to calculate an absorbed dose of water for 40–300 kVp X-rays [54,55,56]. Far too many publications fail to provide sufficient detail to enable replication of radiation delivery, and even fewer provide details regarding the frequency and type of calibration performed [57]. At our institution, monthly quality assurance (QA) is performed by a member of the UW Calibration Lab using a custom-built compact 4 × 4 cm phantom and Gafchromic EBT3 film which has known spatial precision and relatively flat energy response [58]. At a minimum, we suggest that monthly QA be performed to ensure the dose rate is correct and, for small animal image-guided systems, to confirm alignment of the beam with the isocenter. We prefer to use experiment-specific phantoms and thermoluminescent detectors to validate the dose for each experimental model.

There is a tremendous number of different radiation dose schemes that have been utilized for in vivo models (Table 1). We try to utilize clinically relevant schemes such as 2 Gy per day, 5 days per week, for most studies of radiation sensitivity in which we monitor tumor size. These choices must be balanced with the potential toxicity of treatment and the anticipated longevity of the animal. A shorter endpoint should be prioritized in humanized animals due to the potential of graft versus host disease [50,51]. Higher radiation doses per fraction are used when specific questions require an alternative approach. The majority of our studies monitor tumor size over time, but on occasion we perform TCD50 assays aimed at determining the radiation dose required to control half of the tumors [59,60]. This approach can be pursued either by delivering different numbers of 2 Gy radiation fractions or by keeping the total fractions consistent and varying the dose per fraction (for example, see [7]).

### Challenges in Drug and Radiation Delivery

Over 60% of head and neck cancer patients receive chemotherapy and/or radiation during their lifetime. In vivo PDX models provide an opportunity to study the effects of both of these treatments simultaneously, mimicking clinical practice. The ease of studying drug or radiation sensitivity in vitro has allowed for the discovery of chemotherapeutics and molecularly targeted agents that sensitize cells to RT [61,62]. These studies are then translated into murine models with varying rates of success and, unfortunately, these studies are rarely translated to clinic, with a disappointing number of clinical advances [57,62,63,64]. Hypotheses explaining this divide include (1) the disparity in tumor microenvironment (TME) between animal models and human tumors; (2) PK/PD challenges limiting drug availability; (3) failure to confirm target inhibition; (4) unanticipated overlapping toxicities; (5) imprecise and inaccurate RT dose delivery impairing interpretation of the data; (6) testing in a limited number of cell lines; and (7) failure to test with RT.

The tumor stromal microenvironment plays an important role in tumor viability, cell signaling, and resistance to treatment. Patient tumors established as PDXs can retain their original stromal composition, allowing for more clinically relevant studies, however, this is usually only maintained for two to three passages, after which the mouse stroma dominates [65]. Therefore, creating expansions and banking early passage numbers is often recommended. Tumor vascularization is also a significant physiological variable as this affects drug distribution, availability, hypoxia, and tumor cell growth and death. While it is much easier to control drug concentration in vitro, this is not as straightforward in murine models. An important consideration is drug availability and intra-tumoral concentrations. Most research groups utilize published drug dosing regimens, but intra-tumoral concentrations are rarely reported or validated, likely leading to highly variable drug concentrations among studies. Dosing schedules are likely to differ between mouse and human studies as well. Importantly, drug doses in combined chemoradiation studies are also rarely assessed. Additionally, there can be significant differences in how strains of mice metabolize drugs, leading to dose regimens that are effective in one strain but inappropriate for another strain [66,67,68]. Robust knowledge of effective drug concentrations in PDX models compared to patients is lacking, and may certainly affect study results.

These clear challenges in drug delivery do not apply to radiation therapy, however, as dosing is administered externally and occurs homogeneously in all cases provided the tumors are of similar size and appropriate quality assurance has been performed. Radiation administered to PDXs can be given in 2 Gy daily fractions—exactly how patients are treated in the clinic. However, the total radiation dose to PDX models is generally less than the dose used to treat patients, as this would be very time intensive and may lead to ulceration in the mouse. For HNC PDXs, we use 10 Gy in five 2 Gy fractions, which are administered every day. This dosing regimen can slow the growth of radiosensitive tumors and has been shown to recapitulate the radiation sensitivity of HPV-positive and HPV-negative cell lines observed in vitro [26].

## 4. Experimental Design

Estimation of the number of subjects required to answer an experimental question is an important step in planning a study. On one hand, an excessive sample size can result in a waste of animal life, lab resources, and personnel time. However, underestimations of sample sizes are also wasteful, since an insufficient sample size has a low probability of detecting a statistically significant difference between groups, even if a difference really exists. There is no magic solution to decide the size of treatment groups for studies of radiation and/or chemotherapy in xenografts. We work closely with our collaborating statisticians to analyze variability in tumor growth rates in order to identify a proper sample size. Most commonly, we start with groups of 12–15 animals with the expectation that 10%–20% of tumors will either not develop or will grow much faster or slower than the average. This allows us to exclude outliers at the start of treatment.

We also include additional animals in each group that can be euthanized early during treatment to provide tissue to assess drug concentrations, target inhibition, or investigate molecular mechanisms. Three animals per condition are used to provide the biologic replicates of the endpoint. To be clear, this means that an experiment testing the addition of a drug to radiation involves four treatment groups (i.e., mock, drug, RT, drug + RT) and up to 60–72 animals in total.

For the majority of experiments that utilize flank xenografts, we use tumor volume as our endpoint, while for orthotopically implanted tumors we typically use a surrogate of survival (loss of >20% body weight from the baseline). Animals that are euthanized at early time points to support biologic investigations are not included in survival analyses, but are used in tumor volume analysis. As stated above, once PDX tumors reach 200 mm^3^, they are irradiated and tumor volume is measured every 2–3 days for approximately 30–50 days.

## 5. Statistical Analysis

The statistical methods applied to analyze tumor volume (longitudinal data) from PDXs typically involve using linear mixed-effects models [69]. The fixed-effects model matrix is parameterized to reflect the experimental design (e.g., to compare one drug vs a control, or a combination of two drugs vs a gold-standard treatment, or comparing PDX implantation sites, etc.), while the random effects structure is often specified (data allowing) to accommodate intra-mouse variation and temporal correlation (random slope and intercept). If the regression model assumptions are not adequately met, both variable transformations (usually log-transforming the data) as well as generalized linear models can be entertained. Models are generally fit using restricted maximum likelihood estimation (REML) and model inference is typically conducted using parameter-level confidence intervals obtained by bootstrapping and p-values approximated using the Kenward–Roger method. Limitations to this approach include the inability to process censored data, and the assumption of linearity (on the natural scale). Other complications include adequately modeling the cross-generational correlation inherent in PDXs.

For experiments where the primary outcomes of interest are time-to-event data (such as tumor doubling, animal death, loss of % body weight, etc.), the semi-parametric Cox proportional-hazards regression model is typically preferred, as it can handle multiple predictors and allow for covariates to be included [70]. Between-group differences in hazard functions are assessed using the score test (equivalent to the log-rank test) and potential confounding effects can be adjusted via model parameterization. 

## 6. Conclusions

In this era of personalized cancer therapy, head and neck cancer PDXs offer the opportunity to directly test drug and radiation combinations on patient tumors, while allowing for biologic and genetic correlative studies. Importantly, PDXs have been shown to recapitulate the original patient tumor with regard to histology and molecular profiles, including the expression of viral oncogenes in the case of HPV-positive HNC, enabling biologically and clinically relevant studies. Head and neck PDXs can be grown heterotopically or orthotopically and it is currently unclear if one model is superior to the other. These tumors can be treated with both chemotherapy and radiation, mimicking patient treatment paradigms and enabling a comparison of treatment regimens. However, the limitations of this model must not be overlooked, and include differences in the tumor microenvironment due to the presence of mouse stromal tissue, difficulty controlling intra-tumoral drug concentrations, and the lack of a functional immune system. Given the integral role of the immune system in treatment response and the burgeoning field of immunotherapy, it will be essential to discover new ways to study PDXs in immunocompetent models.

## Figures and Tables

**Table 1:** Common radiation doses, fractionation schemes and measured endpoints.

Radiation Dose	Fractions	Schedule	Chemotherapy	Endpoint
2–3 Gy/fraction	5–10 fractions	daily × 1–2 weeks	+/−	Tumor growth delay, growth rate, time to tumor doubling
2 Gy/fraction	25–35 fractions	daily × 5–7 weeks	+/−	Cure rate, tumor control dose—50%
5–10 Gy/fraction	1–8 fractions	daily, 3 times/week	+/−	Tumor growth delay, growth rate, time to tumor doubling, cure rate, tumor control dose—50%
